# Novel Bispidine-Monoterpene Conjugates—Synthesis and Application as Ligands for the Catalytic Ethylation of Chalcones

**DOI:** 10.3390/molecules26247539

**Published:** 2021-12-13

**Authors:** Evgeniy V. Suslov, Konstantin Y. Ponomarev, Oxana S. Patrusheva, Sergey O. Kuranov, Alina A. Okhina, Artem D. Rogachev, Aldar A. Munkuev, Roman V. Ottenbacher, Alexander I. Dalinger, Mikhail A. Kalinin, Sergey Z. Vatsadze, Konstantin P. Volcho, Nariman F. Salakhutdinov

**Affiliations:** 1 Department of Medicinal Chemistry, N.N. Vorozhtsov Novosibirsk Institute of Organic Chemistry SB RAS, Lavrent’ev Av., 9, 630090 Novosibirsk, Russia; suslov@nioch.nsc.ru (E.V.S.); ponomarev@nioch.nsc.ru (K.Y.P.); bawos@nioch.nsc.ru (O.S.P.); skuranov@nioch.nsc.ru (S.O.K.); aokhina@nioch.nsc.ru (A.A.O.); rogachev@nioch.nsc.ru (A.D.R.); amunkuev@nioch.nsc.ru (A.A.M.); anvar@nioch.nsc.ru (N.F.S.); 2 Faculty of Natural Sciences, Novosibirsk State University, Pirogov Str., 2, 630090 Novosibirsk, Russia; 3 Boreskov Institute of Catalysis SB RAS, Lavrent’ev Av., 5, 630090 Novosibirsk, Russia; ottenbacher@catalysis.ru; 4 Chemistry Department, Lomonosov Moscow State University, Leninskie Gory, MSU, 1-3, 119991 Moscow, Russia; dal1995@mail.ru (A.I.D.); chem.kalinin@gmail.com (M.A.K.)

**Keywords:** bispidines, monoterpenoids, catalysis, dialkylzincs addition, chalcones, natural compounds, chiral ligands, ethylation, nickel complexes

## Abstract

A number of new chiral bispidines containing monoterpenoid fragments have been obtained. The bispidines were studied as ligands for Ni-catalyzed addition of diethylzinc to chalcones. The conditions for chromatographic analysis by HPLC-UV were developed, in which the peaks of the enantiomers of all synthesized chiral products were separated, which made it possible to determine the enantiomeric excess of the resulting mixture. It was demonstrated that bispidine-monoterpenoid conjugates can be used as the ligands for diethylzinc addition to chalcone C=C double bond but not as inducers of chirality. Besides products of ethylation, formation of products of formal hydrogenation of the chalcone C=C double bond was observed in all cases. Note, that this formation of hydrogenation products in significant amounts in the presence of such catalytic systems was found for the first time. A tentative scheme explaining the formation of all products was proposed.

## 1. Introduction

The presence of two spatially close-up nitrogen atoms in the structure of the 3,7-diazabicyclo[3.3.1]nonane (bispidine) framework makes bispidines promising bidentate ligands for transition metal complexes [1,2,3]. The possibility of obtaining different bispidine derivatives with substituents at both the carbon scaffold and the nitrogen atoms [4], allows one to vary the rigidity of bispidine core and electronic properties of bispidine complexes, as well as steric accessibility of possible catalytic metal sites of bispidine complexes. This in turn may lead to obtaining catalytic systems with required catalytic properties [5]. It should be noted that the conformational rigidity of the bispidine core determines the volume parameters of coordination space, firstly N…N distance. This parameter mainly determines the selectivity of complex formation and the stability of the resulting complexes. Thus, relatively small ions of transition metals like Cu(II), Ni(II), Pd(II), Zn(II) are the most advantageous to form such compounds [6]. Complexes of substituted bispidines with transition metals have been investigated in Michael [7] and Henry reactions [8], deprotonation/electrophilic addition [9], oxidative kinetic separation of secondary alcohols [5], hydrogenation of double bonds [5], addition of diethylzinc to aldehydes [5,10] and others.

**Scheme 1:**
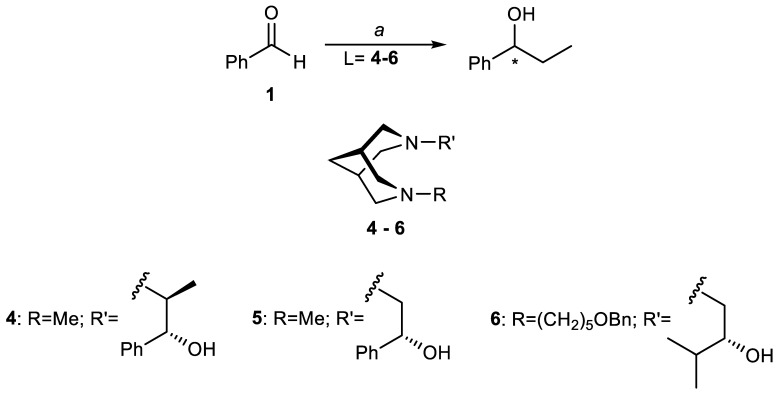
Reactions of diethylzinc with benzaldehyde employing bispidines as ligands. Reaction conditions: *a*—Et_2_Zn, **4–6** (3–10 mol%), toluene, −78 °C up to 0 °C or 0 °C up to r.t. Here and further in the article: the asterisk denotes a chiral center of a molecule.

**Scheme 2:**
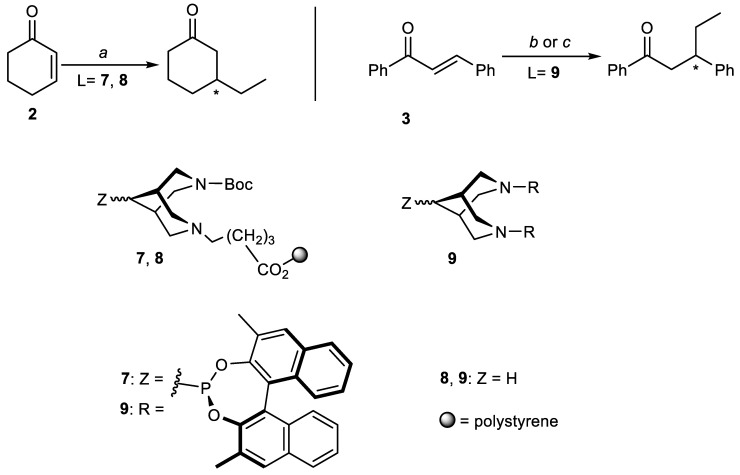
Examples of some reactions of diethylzinc with α,β-unsaturated carbonyl compounds employing bispidines as ligands. Reaction conditions: *a*—Cu(OTf)_2_ (3 mol%), **7**, **8** (3.3 mol%), toluene, CH_2_Cl_2_, r.t., then **2**, Et_2_Zn, 0 °C; *b*—Ni(acac)_2_ (7 mol%), Et_2_Zn, **1**, **2**, **9** (13–16 mol%), MeCN, −30 °C; *c*—Cu(OTf)_2_ (3 mol%), **9** (3.3 mol%), toluene, CH_2_Cl_2_, r.t., then **3**, Et_2_Zn, −15 °C.

Various bispidine derivatives were successfully used as ligands catalyzing the interaction of diethylzinc with benzaldehyde **1**, cyclohexenone **2** and chalcone **3** [5,10,11,12,13] (Scheme 1 and Scheme 2). Thus, it was shown [11,14,15] that the use of bispidines with *N*-alkyl substituents or bispidines with *N*-β-hydroxyalkyl substituents as a component of catalytic system led to the product of benzaldehyde ethylation with good yields (up to 97%) and enantiomeric excess (*ee*) up to 96%, while the best results were obtained with ligands **4–6** (Scheme 1).

The possibility of utilizing bispidine ligands in the nickel-catalyzed Michael addition of diethylzinc to the chalcone double bond has also been investigated [11,15]. Compounds **4** and **5** proved to be the most efficient in this transformation: yields of the desired product were up to 92% with *ee* up to 79%.

The copper-catalyzed Michael addition of Et_2_Zn to cyclohexenone **2** in the presence of several BINOL modified bispidines immobilized on polystyrene was studied [12,13] (Scheme 2). The best results were obtained with ligands **7** and **8**: yields exceeded 90% with *ee* up to 67%. Bispidines modified by BINOL at both nitrogen atoms were also investigated in the interaction of chalcone **3** with diethylzinc [16] in the presence of copper triflate. The use of ligand **9** (Scheme 2) allowed one to obtain the desired product in almost quantitative yield (98%) and *ee* 78%.

It should be noted here that the addition reactions of diethylzinc to enones require not only the presence of a suitable ligand but also the presence of a nickel or copper salts.

**Scheme 3:**
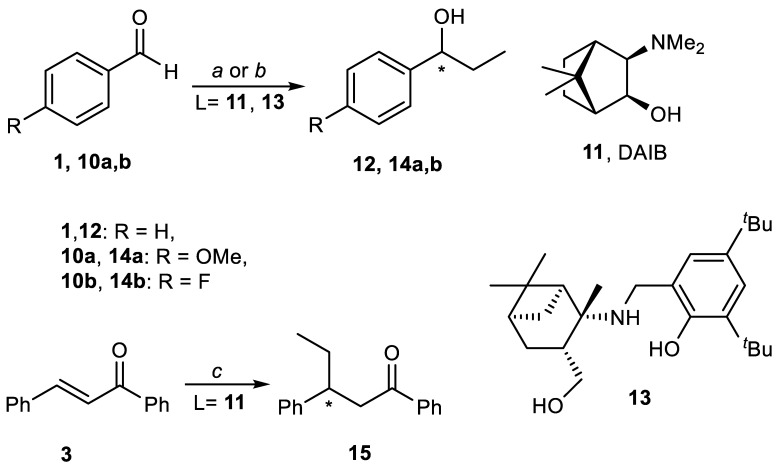
Reactions of Et_2_Zn with benzaldehydes **1**, **10** and chalcone **3** using ligands based on natural compounds. Reaction conditions: *a*—11 (2 mol %), Et_2_Zn with 1 (1.2: 1 molar ratio), toluene, 0 °C; *b*—**1**, **12a**,**b**/Et_2_Zn/Ti(O*i*-Pr)_4_/**13** molar ratio was 1/1.1/1/0.1, hexane, 0 °C; *c*—Ni(acac)_2_ (7 mol%), **11** (16 mol %), MeCN, hexane, −30 °C.

A number of natural compound derivatives, including monoterpenoids, can also be successfully used as ligands in the interaction of diethylzinc with benzaldehydes **1**, **10a**,**b** and chalcone **3 [17,18,19,20,21,22,23,24,25]**. One of the first highly enantioselective ligands for ethylation of benzaldehyde with diethylzinc was (-)-3-exo-(dimethylamino)isoborneol (DAIB) **11** (Scheme 3) [17]; its use allowed one to obtain product **12** with *ee* up to 99% and yield up to 98%. Some pinene derivatives such as compound **13** (Scheme 3) have also been used in similar transformations and resulted in the desired alcohols **14a**,**b** in yields up to 77% and ee up to 80% [20]. Compound **11** was also studied as a ligand in nickel-catalyzed reactions of Et_2_Zn with chalcone **3 [21,22]**, in which the desired ketone **15** was formed in a yield up to 90% and *ee* up to 85%. It should be noted that the study of the effect of new chiral ligands on the interaction of diethylzinc with benzaldehyde, cyclohexenone and chalcone in test reactions is often a necessary starting point for the development and optimization of catalytic systems in the approach to more complex and practically significant substrates.

The aim of this work is the synthesis of chiral bispidine derivatives containing monoterpenoid fragments and the study of their use as ligands in the nickel-catalyzed addition of Et_2_Zn to chalcones.

## 2. Results and Discussion

**Scheme 4:**
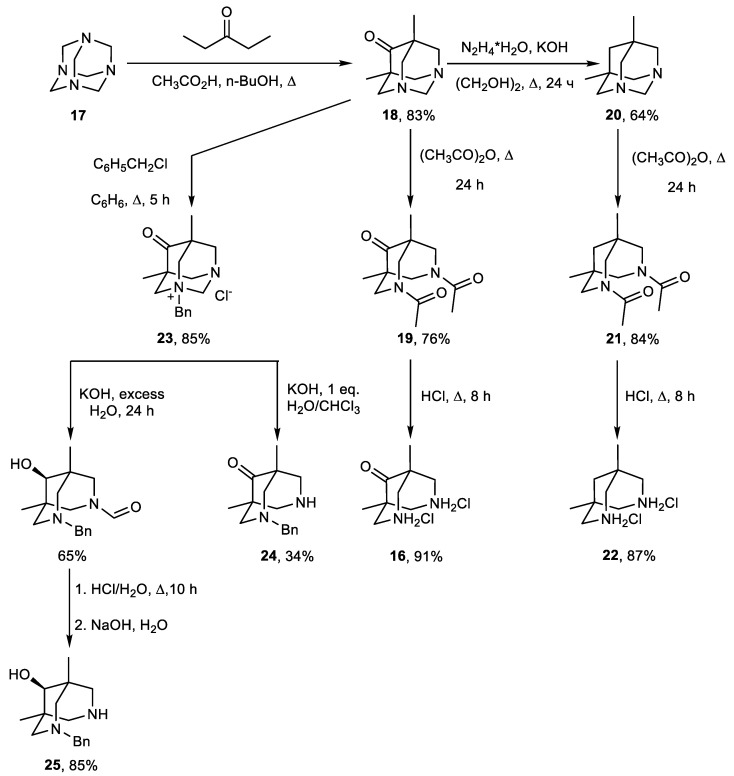
Synthesis of compounds **16**, **22**, **24** and **25**.

The synthesis of the starting 1,5-dimethyl-3,7-diazabicyclo[3.3.1]nonane **16** was carried out by the following procedure (Scheme 4) [26]. For this purpose, 1,3-diazaadamantan-6-one **18** was obtained from hexamethylenetetramine **17** and its acylation with acetic anhydride gave acetyl derivative **19**. Subsequent acidic hydrolysis with aqueous hydrochloric acid solution yielded corresponding salt of bispidinone **16**.

For synthesis of bispidines lacking keto-group at the 9th position, diazaadamantanone **18** was reduced with hydrazine hydrate in alkaline medium according to the procedure [27], which led to formation of 1,3-diazaadamantane **20**. Its acylation with acetic anhydride followed by hydrolysis with hydrochloric acid of resulting amide **21** gave bispidine **22** (Scheme 4).

**Scheme 5:**
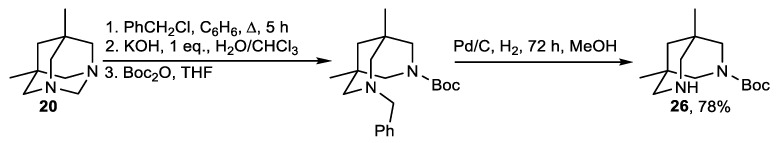
Synthesis of compound **26**.

By the interaction of diazaadamantanone **18** with benzyl chloride followed by KOH treatment of the resulting quaternary ammonium salt **23**, monobenzyl-substituted bispidinone **24** was synthesized (Scheme 4) [8,28]. Hydrolysis of compound **23** with excess of alkali followed by acid treatment led to the formation of bispidinol **25**. Compound **26** was synthesized from diazaadamantane **20** by its subsequent interaction with benzyl chloride, treatment with potassium hydroxide in mixture H_2_O/CHCl_3_, introduction of *tert*-butoxycarbonyl protecting group and removal of benzyl fragment by Pd/C-H_2_ system (Scheme 5).

**Scheme 6:**
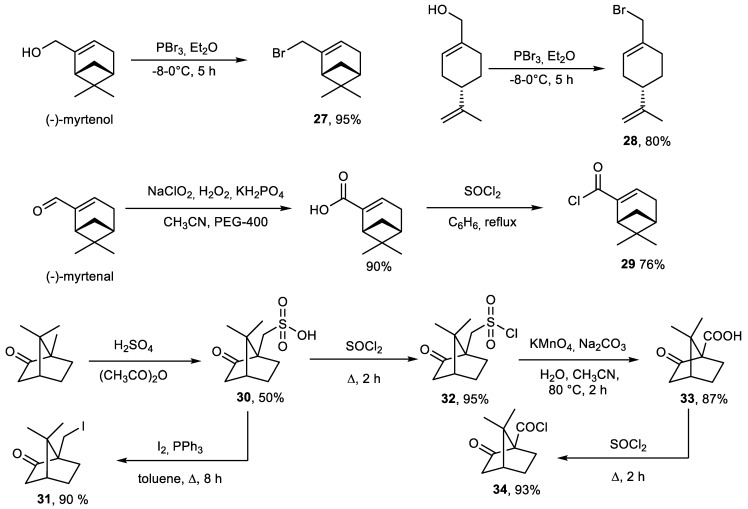
Synthesis of compounds **31**–**34**.

Bulky bicyclic molecules, i.e., (-)-myrtenol and (+)-camphor, and also monocyclic (*S*)-perillyl alcohol were chosen as terpene fragments for chiral ligands. Bromo-derivatives **27** and **28** (Scheme 6) were prepared by interaction of the corresponding alcohols with phosphorus tribromide under cooling according to the procedure [29]. The acid chloride **29** was synthesized by oxidation of (-)-myrtenal followed by treatment of the resulting acid with thionyl chloride [26]. Camphorsulfonic acid **30** was obtained from camphor, according to the procedure [30]. By its refluxing in toluene with iodine in the presence of triphenylphosphine, camphor iodine derivative **31** was synthesized [31]. To study the influence of nature of the linker on the result of the catalyst reaction, sulfonyl chloride **32** and ketopinic acid **33** [32] that was transformed into corresponding acid chloride **34** were obtained.

**Scheme 7:**
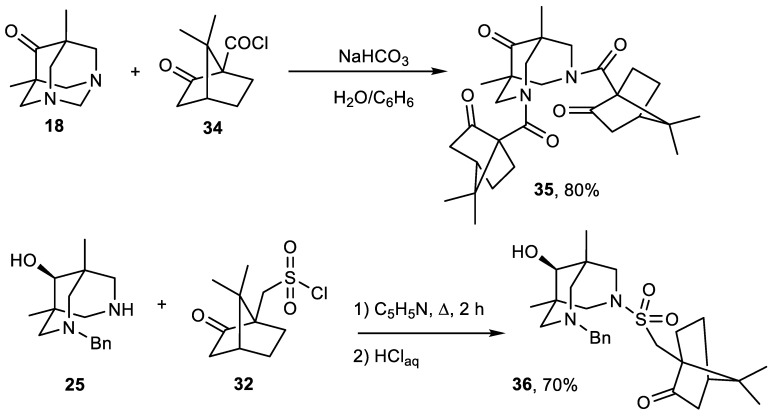
Synthesis of compounds **35** and **36**.

Target conjugate **35** was obtained by acylation of diazaadamantanone **18** with ketopinic acid chloride **34** in two-phase benzene/water system in 80% yield (Scheme 7). The sulfonamide **36** was synthesized from monobenzylsubstituted bispidinol **25** by refluxing in pyridine in 70% yield (Scheme 7).

**Scheme 8:**
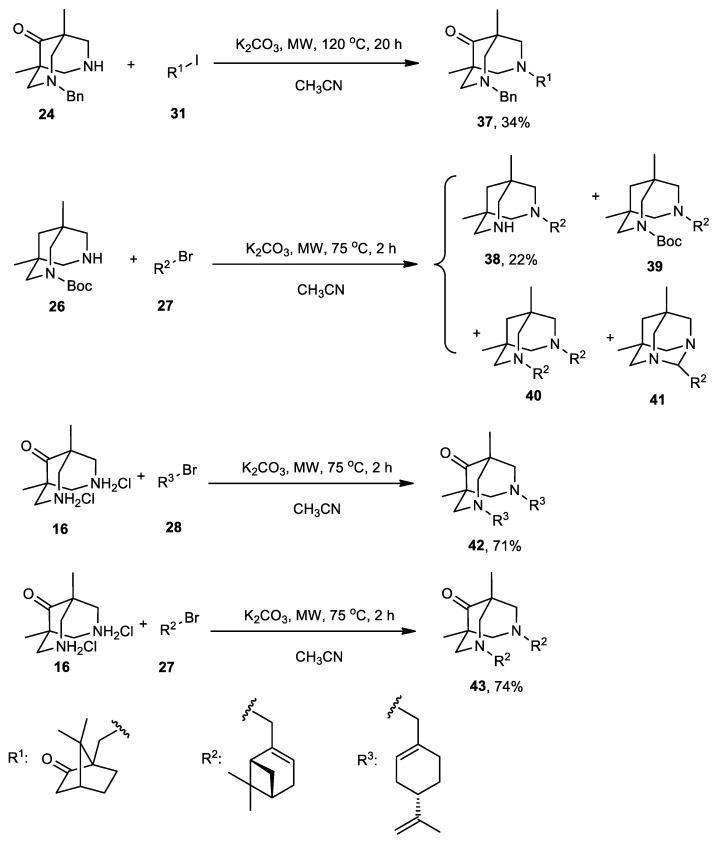
Synthesis of compounds **37**, **38**, **42** and **43**.

Amino derivatives **37** and **38** were synthesized under microwave irradiation (Scheme 8). The product **37** was obtained by the interaction of *N*-benzylbispidinone **24** with camphor iodine derivative **31**. Its formation required more severe conditions (increase of reaction temperature from 75 °C to 120 °C and reaction time from 2 h to 20 h) than for the previously described derivatives with pinane framework [8] which seemed to be associated with a higher steric hindrance of reaction center in the molecule of the terpene derivative. Bispidinone **16** without alkyl substituents at the nitrogen atom failed to interact with the camphor iodine derivative **31** under all studied conditions. In the case of the interaction between Boc-substituted bispidine **26** and bromine derivative **27** we observed the formation of *N*- and *N*,*N’*-substituted products **39** and **40** as well as diazaadamantane **41** in addition to compound **38**. Product **38** was isolated in 22% yield, whereas the other compounds formed an inseparable complex mixture.

Previously we obtained disubstituted bispidinone **43** in [8] (Scheme 8). In the present work this compound was also synthesized by interaction of bispidinone **16** with corresponding monoterpenoid bromide in 74% yield.

Thus, we synthesized a number of chiral bispidines containing one or two monoterpenoid fragments.

**Scheme 9:**
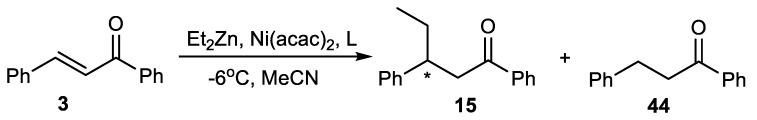
Reactions of Et_2_Zn with chalcone **3** in the presence of ligands containing fragments of monoterpenoids; L = **35**, **38**, **42**, **43**.

The obtained compounds were tested as ligands in the nucleophilic addition reaction of diethylzinc to the chalcone double bond (Scheme 9). This reaction was chosen as a test reaction since the interaction of α,β-unsaturated enones with organozinc compounds allows one to easily synthesize chiral β-substituted carbonyl compounds with a variety of biological activities [33,34,35]. In this kind of transformation, both copper and nickel salts can be used as catalysts [11,15,21,22]. As a component of the catalytic system, we chose Ni(acac)_2_ as a cheaper and more accessible reagent than copper triflate also used in such reactions. It should be noted that in this type of reactions [11,15,21,22] Cu and Ni salts usually lead to similar results.

On the basis of available published data [11,15,21,22], different reaction conditions could be applied. Thus, during the optimization stage we tried to vary solvents (Table 1), temperature (Table 2), ligand amount and reaction time. Hexane, diethyl ether, dichloromethane, acetonitrile were used as solvents; reaction temperature was varied from −20 to 22 °C; ligand amounts from 8 to 24 mol%; reaction time from 1 to 16 h. The catalyst amount of Ni(acac)_2_ was 7 mol% [21,22], the molar ratio chalcone: diethyl zinc was 1:2. Using 1,5-dimethyl-3,7-diazabicyclo[3.3.1]nonan-9-one **42** disubstituted with (*S*)-perillyl alcohol fragments as a ligand, we found that MeCN was the optimal solvent since the yield of compound **15** was the highest and reached 63% after stirring the reaction mixture at −6 °C for 4 hrs. The yields of ketone **15** as well as the conversion of the starting chalcone were determined after their isolation from the reaction mixtures by column chromatography. It should be noted that the reaction of chalcone with diethylzinc did not proceed in Et_2_O (Table 1). The conversion of starting chalcone **3** at −6 °C in acetonitrile was >98%, lowering temperature of reaction to −20 °C results in decreasing the conversion to 77% (Table 2).

It should be noted that in addition to the expected product **15** (Scheme 9, Table 3), ketone **44** is also formed in the reaction mixture in comparable amounts. To optimize the amount of ligand in the reaction of compounds **15** and **44**, we used diamine **42** as a model compound. It was found that 30 min after start of the reaction the maximal ratio of **15** to **44** (according to GC/MS data) was observed when 24 mole percent of the ligand **42** (Table 3) was used with respect to the starting chalcone **15**.

Formation of compound **44** as a by-product of the transformations was observed in [36] where *C_2_*-symmetrical 2,2′-bipyridines were used as the ligands for Michael addition of diethylzinc to chalcone **3** in the presence of Ni(acac)_2_. Ketone **44** was detected by the authors of the work according to ^1^H NMR data in trace amounts. They also proposed a possible mechanism of nickel catalyzed transformations which was similar to that considered in the study [11].

**Scheme 10:**
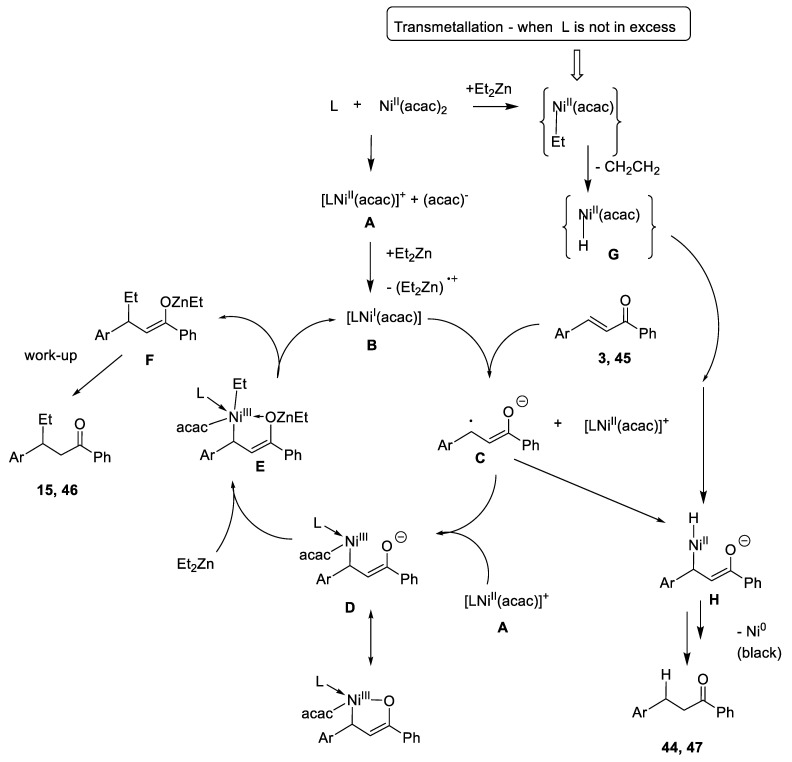
Possible mechanism for the formation of compounds **15**, **46** and **44**, **47**.

Based on the literature data and our own results, a tentative scheme explaining the formation of both products (**15** and **44**) could be proposed (Scheme 10). The main catalytic cycle starts from the formation of [LNi^II^acac]^+^ (**A**) reactive species when the amount of L is enough to complex all the Ni precursors (complex [L_2_Ni]^2+^ is less possible due to the presence of bulky groups in L). The addition of Et_2_Zn leads to the reduction of **A** to form radical particle [LNi^I^acac] (**B**) which reduces the starting chalcone to the radical-anion **C**. Intermediate **A** adds to this radical-anion to form the Ni^III^ product **D**. The latter reacts with Et_2_Zn to form Zn-enolate **E** which undergoes the reductive elimination to form catalytically active **B**. The thus-formed enolate **F** gives rise to the product **15** after work-up.

In the case when amount of the ligand is equal or slightly exceed the amount of the Ni complex, the formation of the required catalytic complex **A** is not fully accomplished due to comparatively less stability of bispidine-Ni complexes than corresponding bispidine-Co or -Cu complexes [37]. This leads to the presence in the reaction mixture the uncomplexed Ni species. Upon addition of Et_2_Zn the transmetallation reaction could occur which in turn would make the β-elimination of ethylene from Ni-Et complex possible. The resulting Ni-H species **G** could compete with the main catalytic pathways (**C** to **E**) and add to the chalcone C=C double bond (intermediate **H**) with subsequent reductive elimination of Ni(0) species. Only in the case when we have a great excess of ligand (24 to 7), the [LNiacac]^+^ is formed completely, and the side-reaction would not compete with the main one.

All the further experiments on the use of monoterpenoid-substituted ligands were performed according to the following procedure: solutions of Ni(acac)_2_ (7 mol% of the chalcone amount) and ligand (24 mol%) were premixed in the required amount of acetonitrile and stirred for 1 h at 80 °C. Then the resulting solution was cooled to room temperature and a solution of chalcone in dry acetonitrile was added, the resulting mixture was cooled to −6 °C and 2 equivalents of 1M Et_2_Zn solution in hexane were added under argon, the final mixture was stirred for required time at −6 °C. The composition of the reaction mixtures was analyzed by gas chromatography with mass spectrometric detection (GC/MS) as well as by high performance liquid chromatography (HPLC) on a chiral sorbent column. For the assignment of peaks in the chromatograms, 1,3-diphenylpropane-1-one **43** and racemic 1,3-diphenylpentane-1-one **14** prepared according to procedures [38,39] were analyzed. The ratio of the reaction products **15** and **44** as well as the starting chalcone **3** was determined by HPLC-UV. For this purpose, the UV spectra of each compound were first recorded and their extinction coefficients at the absorption maximum were determined. After integrating the peaks in the chromatograms their areas were obtained, and the ratio of the components of the reaction mixture was calculated.

To develop a method for determining the enantiomeric excess, a number of preliminary experiments were carried out using racemic 1,3-diphenylpentane-1-one **15**. In this work, a column packed with chiral Lux Cellulose-1 stationary phase was used which is a cellulose phenylcarbamate derivative. It was found that upon isocratic elution of compound **15** with a hexane-isopropanol mixture containing more than 10% of the latter, the substance eluted virtually immediately after the column dead volume and no separation of enantiomers occurred. Reducing the isopropanol concentration in the eluent to 1–2% (*v*/*v*) resulted in almost complete separation of the enantiomers peaks, while reducing the isopropanol concentration in the eluent to 0.8% by volume allowed satisfactory peak resolution without a significant increase in overall analysis time. In a study of the effect of column temperature on the separation of enantiomers, it was found to be more efficient at 20 °C than at higher temperatures. Reducing the temperature to 15 °C increased the pressure in the system by increasing the viscosity of the eluent but did not provide a more efficient separation of the enantiomers during analysis. No elution of the starting chalcone **3** as well as its reduction product 1,3-diphenylpropan-1-one **44** occurs during isocratic elution using 0.8% isopropanol in hexane. In a series of experiments, it was found that both substances were eluted when the column is washed with a hexane/isopropanol 80:20 (*v*/*v*) mixture. In order to completely characterize the reaction mixtures, a gradient consisting of two isocratic steps was used for analysis: elution with a 0.8% isopropanol/hexane system providing enantiomeric separation, followed by an abrupt switch to a 20% isopropanol/hexane system allowing the starting chalcone **3** and reduction product **44** to be identified during a single chromatographic run. As a result, a method was developed that allowed not only the determination of the enantiomeric excess, but also the ratio of the reaction products to the starting chalcone content. An example of a chromatogram of the reaction mixture is shown in Figure S9.

The elaborated chromatographic analytical procedure allowed us to study content of the starting compound and the products during the reaction. For example, in Table 4 the content in the reaction mixtures of compounds **3**, **15** and **44** after 15, 30 and 60 min after the start of the reaction is given.

The presented data show that the catalytic system containing ligand **43** has the highest activity after 15 min, while the benzyl-substituted bispidine **37** is the least active. At 30 min after the start of the reaction the lowest content of chalcone is observed for ligands **42**, **43** and **37**, and after 1 h for ligands **35**, **42** and **43**. The highest content of the alkylation product of compound **15** (>60%) was found when diamines **42** and **43** were used as ligands. In all cases, except compound **37**, the formation of compound **44**, a formal hydrogenation product of the chalcone double bond, is observed 15 min after the start of the reaction. The analysis of the ratio **15**/**44** shows that 60 min after the start of the reaction the ratio is lowest (0.63) for diamide **35** and highest for diamine **43** (2.07). The same ratio is comparable for bispidines **36** and **42** (~1.8) and **37** and **38** (~0.8) 1 h after the start of the transformation. We noted that keeping compound **15** under reaction conditions for 60 min using diamine **43** as a ligand did not lead to the formation of compound **44**. In all cases in which compounds **35–38**, **42** and **43** are used as ligands we observe the formation of a virtually racemic ketone **15**, with enantiomeric excess not exceeding 5%. It should be noted that the exception of Ni(acac)_2_ from the catalytic system led to a dramatic decrease in the reaction rate. Thus, while ligand **42** and diethylzinc were used without the addition of Ni(acac)_2_, the content of compound **15** was 9% after 6 h.

**Scheme 11:**
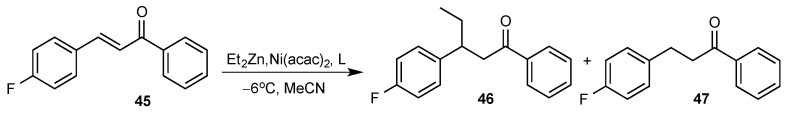
The addition of diethylzinc to chalcone **45**; L = **35**, **37** and **43**.

The fluorinated chalcone **45** was also used as a substrate for the catalytic diethylzinc conjugate addition (Scheme 11, Table 5). The data in Table 5 show that ligand **43** was the most active in these transformations: full conversion of chalcone **45** was observed already after 30 min, whereas for the other bispidines it was less than 50% at this time. As in the case of chalcone **3**, ligand **37** did not lead to the formation of the reduction product **47** after 15 min, and the study of the reaction mixtures after 30 min showed that the rate of hydrogenation product formation was the lowest for diamine **37**. Comparative analysis of the data for chalcone **3** and **45** showed that in the case of the fluorinated substrate all ligands led to a lower content of hydrogenation product **47** than that observed for compound **44**. As in the case of compound **15**, ketone **46** was formed almost completely as a racemic mixture.

The results shown in Table 4 and Table 5 could be qualitatively explained by the comparison of the ligand ability to form stable Ni^II^ complexes and the ratio between desired product **15** and by-product **44**. For example, while the di-tertiary diamine **42**, which should form stable complexes, gives rise to the highest amount of ethylated product **15**, the diamide **35** leads to the highest amount of the hydrogenated product **44**. These findings are in excellent agreement with the proposed catalytic scheme suggested in Scheme 10.

It should be noted that the exception of Ni(acac)_2_ from the catalytic system led to a dramatic decrease in the reaction rate. Thus, while ligand **42** and diethylzinc were used without the addition of Ni(acac)_2_, the content of compound **15** was 9% after 6 h.

In summary, a number of new chiral bispidines combined with monoterpenoid fragments have been synthesized. The study of their properties as the participants of the catalytic system of Ni(acac)_2_/Et_2_Zn addition of diethylzinc to the chalcone double bond revealed that they can be used as ligands for this type of transformation but not as inducers of chirality. The formation of hydrogenation products of the chalcone double bond in significant amounts in the presence of such systems has been demonstrated for the first time.

## 3. Experimental Section

In this work benzylideneacetophenone (chalcone) (Sigma Aldrich, ≥97%) and diethylzinc, 1M solution in hexane (Sigma Aldrich, St. Louis, MO, USA) were used. All other chemicals were of commercial grade and used without further purification unless otherwise stated. The solvents were freshly distilled.

The ^1^H and ^13^C NMR spectra of the obtained reaction mixtures and individual compounds were carried out with Bruker AV-400 (^1^H: 400.13 MHz, ^13^C: 100.51 MHz) and Bruker AV-300 (^1^H: 300.13 MHz, ^13^C: 75. 47 MHz) as well as obtained ligands as individual compounds with Bruker DRX-500 (^1^H: 500.13 MHz, ^13^C: 125.76 MHz) and Bruker Avance-III 600 (^1^H: 600.30 MHz, ^13^C: 150.95 MHz) spectrometers in CDCl_3_ solution. Chloroform solvent signals (δ_H_ 7.24 ppm, δ_C_ 76.90 ppm) were used as internal standard; *J* in Hz. The structure of the obtained compounds was determined on the basis of analysis of ^1^H NMR spectra involving ^1^H-^1^H double resonance spectra, ^13^C NMR spectra carried out in *J*-modulation mode (JMOD), two-dimensional heteronuclear ^13^C-^1^H correlation spectra at direct (^13^C-^1^H COSY and HSQC, ^1^*J*_C-H_ 135 Hz and 145 Hz, respectively) and long-range spin-spin interaction constants (COLOC and HMBC, ^2,3^*J* 10 and 7 Hz, respectively), and two-dimensional homonuclear ^1^H-^1^H correlation spectra (COSY, NOESY). The elemental composition was determined on the basis of mass spectra carried out with a Thermo Scientific DFS spectrometer in full scan mode in the range of 0–500 *m*/*z*, electron impact ionization 70 eV at direct sample input. The specific rotation was determined with a polAAr 3005 polarimeter for solutions of the compounds in CHCl_3_. A Monowave 300 microwave reactor (Anton Paar, Graz, Austria), was used in the work.

Reaction mixtures and isolated products were analyzed by HPLC with UV detection with a chromatograph Maestro (Interlab, Moscow, Russia) equipped with a high-pressure gradient pump, automatic sampler, column thermostat and UV detector with diode sensor. A Lux Cellulose-1 sorbent column (Phenomenex, Torrance, CA, USA), particle diameter 5 µm, column size 4.6 × 250 mm, equipped with a pre-column with the same sorbent and thermostatted at 20 °C was used as chiral selector. Hexane-isopropanol mixture (99.2:0.8, *v*/*v*) was used as eluent A and isopropanol as eluent B. The shape of the gradient was in the following way: 0 min—0% B; 7 min—0% B, 7.1 min—20% B; 12 min—20% B; the flow rate was 1.5 mL/min. The column was then equilibrated to perform the following analysis. A sample of the reaction mixture was evaporated to dryness in a stream of air if necessary, dissolved in 1 mL of hexane and analyzed; the sample volume was 10 µL. The detection was done at wavelengths of 206, 210, 220 and 260 nm and the full UV spectrum in the range of 200–400 nm was recorded. The chromatograph was controlled, data collected and processed using Clarity 8.2 software (DataApex, Prague, Czech Republic).

The conversion of the reaction was calculated from the ratio of the peak areas of the compounds in the chromatogram using the extinction coefficients measured for each substance individually by UV spectrum at 206 nm and in the corresponding solvent (0.8% or 20% isopropanol in hexane depending on the elution of the substance in the analysis).

### 3.1. General Procedure for Et_2_Zn to Chalcone Addition in the Presence of Ni(acac)_2_ and Bispidine Derivatives Containing Monoterpenoid Substituents

To a solution of 2.4 mmol (24 mol%) of monoterpenoid substituted bispidine, a solution of 0.7 mmol (7 mol%) of Ni(acac)_2_ was added. The mixture was kept at 80 °C for 1 h, then cooled to room temperature and a solution of 10 mmol chalcone was added, then the reaction vessel was purged with argon. The mixture was transferred to a thermostat, cooled to −6 °C and left to stand for 20 min. 200 µL (2 eq, 20 mmol) of 1 M Et_2_Zn solution in hexane was injected through the septa and the mixture was kept in a shaker at −6 °C for the required time. Then the reaction mixture was treated with 2 mL 10% HCl solution, the aqueous layer was separated and extracted with methylene chloride. The organic phases were combined and dried over magnesium sulfate. The dried organic phase was filtered through a silica gel layer, the solvent was evaporated.

### 3.2. Synthesis of a Racemic Mixture of 1,3-Diphenylpentane-1-One ***15***

To a mixture of 104 mg (0.5 mmol) of chalcone **3** and 1.3 mg of NiCl_2_ (2 mol%, 0.01 mmol) was added 2 mL of dry CH_2_Cl_2_. The mixture was heated to 40 °C and 1.0 mL (2 eq, 1.0 mmol) of 1M Et_2_Zn solution in hexane was added for 10 min and allowed to stand for another 10 min. It was diluted with 4 mL ethyl acetate, 7 mL of saturated aqueous NH_4_Cl solution was then added and stirred for 1 h. The aqueous phase was separated and washed with dichloromethane (3 × 8 mL). The organic phases were combined and dried over Na_2_SO_4_. After the solvent was evaporated the product was isolated by column chromatography. The conversion of the starting compound was 100%, the yield of compound **15** was 114 mg (96%). The ^1^H NMR spectrum of compound **15** corresponded to the literature data [38].

### 3.3. Synthesis of Ligands

**Figure 1:**
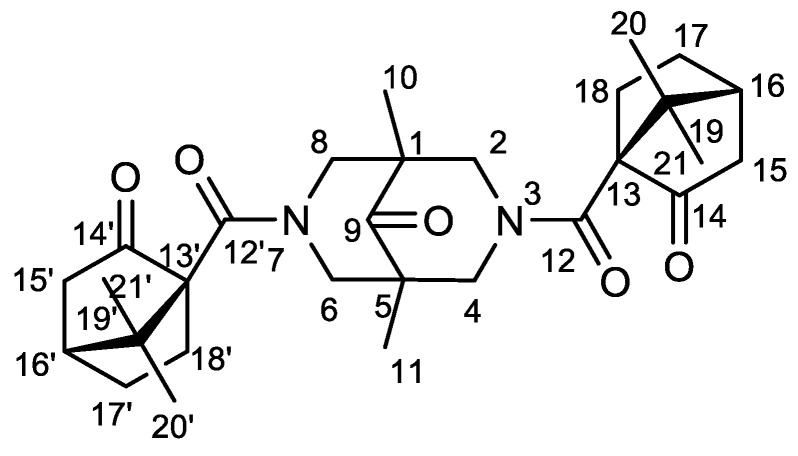
The structure of compound **35** with atom numbering scheme.

(1*S*,1′*S*,4*R*,4′*R*)-1,1′-(1,5-Dimethyl-9-oxo-3,7-diazabicyclo[3.3.1]nonane-3,7-diyl)bis(oxomethylene)bis(7,7-dimethylbicyclo[2.2.1]heptan-2-one) (**35**, Figure 1).

To a mixture of 216 mg (1.2 mmol) diazaadamantane **18** and 202 mg (2.4 mmol) of NaHCO_3_ in 14 mL of benzene and 4 mL of water was added 482 mg (2.4 mmol) of acid chloride **34** in 2 mL of benzene for 10 min. The mixture was allowed to stand for 6 h. The aqueous phase was separated, washed with benzene; the organic phases were combined, the solvent was evaporated. After chromatographic column 240 mg of the product (80%) were isolated.

^1^H NMR (600 MHz, CDCl_3_, δ, ppm, *J*, Hz; for the numbering of the atoms, see Figure 1): 0.99 (s, C^10^H_3_, C^11^H_3_), 1.12 (s, 3H, C^20^H_3_ or C^21^H_3_), 1.20 (s, 3H, C^20^H_3_ or C^21^H_3_), 1.44–1.53 (m, 2H, C^17^H, C^17′^H), 1.85–1.95 (m, 4H, C^17′^H’, C^17′^H’, C^18^H, C^18′^H), 1.99–2.16 (m, 4H, C^15^H, C^15′^H, C^16^H’, C^16′^H’,), 2.32–2.46 (m, 4H, C^15^H’, C^15′^H’, C^18^H’, C^18′^H’), 2.87, 3.37, 3.90, 4.79 (d, 8H, all ^2^*J* = 14.15, C^2^H_2,_ C^4^H_2_ C^6^H_2_ C^8^H_2_).

^13^C NMR (600 MHz, CDCl_3_, δ, ppm): 19.46, 21.37 (q, C20, C20′, C21, C21′), 20.05 (q, C-10, C-11), 26.37 (t, C17, C17′) 28.56 (t, C18, C18′), 42.28 (d, C16, C16′), 43.18 (s, C19, C19′), 44.24 (t, C15, C15′), 46.80 (s, C1, C5), 55.40 (t, C2, C4, C6, C8), 77.16 (s, C13, C13′), 165.36 (s, C12, C12′), 218.34 (s, C14, C14′), 215.70 (s, C9).

$${\left[\alpha \right]}_{D}^{27}=-26.50\;(C=0.4,\;\mathrm{MeOH})$$

**Figure 2:**
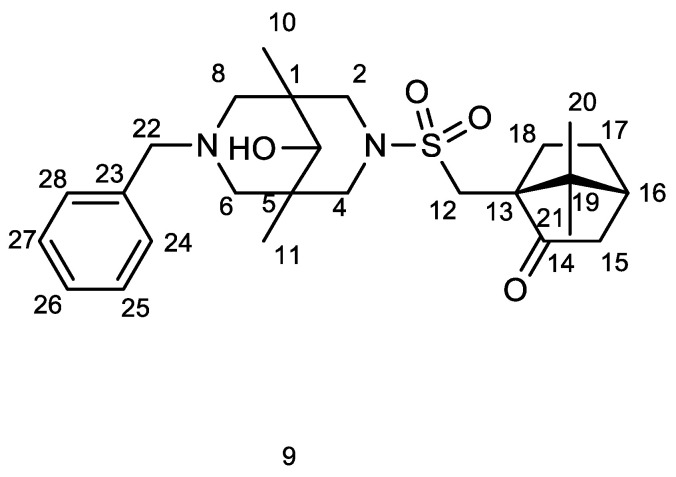
The structure of compound **36** with atom numbering scheme.

Found [*M^+^*]: 496.2937. C_29_H_40_N_2_O_5_^+^. Calculated [*M^+^*]: 496.2933.

(1*S*,4*R*)-1-((7-Benzyl-9-hydroxy-1,5-dimethyl-3,7-diazabicyclo[3.3.1]nonan-3-ylsulfonyl)methyl)-7,7-dimethylbicyclo[2.2.1]heptan-2-one (**36**, Figure 2).

To a solution of 100 mg (0.38 mmol) of bispidinol **25** in 10 mL of pyridine was added a solution of 106 mg (0.42 mmol) of acid chloride **32** in 5 mL pyridine dropwise. The mixture was refluxed for 2 h, pyridine was then evaporated. To the residue, 20 mL of diluted (4%) HCl solution was added and the mixture was stirred for 30 min, then it was extracted with diethyl ether (2 × 20 mL). The organic phase was dried over Na_2_SO_4_ and then evaporated. The resulting mixture was purified by column chromatography on silica gel (3 g of silica gel, eluent n-C_6_H_14_/EtOAc). The desired product was isolated in an amount of 130 mg (yield 70%).

^1^H NMR (600 MHz, CDCl_3_, δ, ppm, *J*, Hz; for the numbering of the atoms, see Figure 2): 0.86 (s, 3H, C^20^H_3_ or C^21^H_3_), 1.02 and 1.03 (s,6H, C^10^H_3_, C^11^H_3_), 1.11 (s, 3H, C^20^H_3_ or C^21^H_3_), 1.38–1.43, 1.55–1.62 (m, 2H, C^17^H, C^18^H), 1.91 (d, 1H, *J* = 18.4, C^15^H), 1.99–2.06, 2.06–2.09 (m, 2H, C^17^H’, C^18^H’), 2.00 (s, 1H, C^16^H), 2.32–2.38 (dm, 1H, *J* = 10.7, C^15^H’), 2.42–2.60 (m, 4H, C^6^H and C^8^H), 2.49 (m, 1H, C^15^H’), 2.96 (s, 1H, C^9^H), 3.34–3.52 (m, 3H, C^22^H, OH), 2.25 (d, 1H, ^2^*J* = 10.7, C^6^H or C^8^H), 2.29–2.34 (m, 1H, C^15^H’), 2.34 (d, 1H, ^2^*J* = 14.4, C^12^H), 2.50 (d, 1H, ^2^*J* = 10.7, C^2^H or C^4^H), 2.55 (d, 1H, ^2^*J* = 10.7, C^2^H or C^4^H), 2.56 (d, 1H, ^2^*J* = 14.4, C^12^H’), 2.94 (d, 2H, ^2^*J* = 10.7, C^6^H’, C^8^H’), 3.13 (d, 1H, ^2^*J* = 10.7, C^2^H’ or C^4^H’), 3.26 (d, 1H, ^2^*J* = 10.7, C^2^H’ or C^4^H’), 3.45–3.52 (m, 2H, C^22^H), 7.20–7.25 (m, 1H, C^26^H), 7.24–7.30 (m, 4H, C^23^H, C^24^H, C^25^H, C^27^H).

^13^C NMR (151 MHz, CDCl_3_, δ, ppm): 46.77, 46.85 (s, C1, C5), 67.63, 68.06 (t, C2, C4), 65.07, 65.17 (t, C6, C8), 79.60 (s, C9), 14.07 (q, C10), 22.48 (q, C11), 52.54 (t, C12), 64.57 (s, C13), 215.49 (s, C14), 43.35 (t, C15), 43.34 (d, C16), 25.28, 26.89 (t, C17, C18), 47.25 (s, C19), 19.66, 19.85 (q, C10, C11), 61.24 (t, C22), 138.04 (s, C23), 128.63 (d, C24, C28), 128.30 (d, C25, C27), 127.10 (d, C26).

$${\left[\alpha \right]}_{D}^{23}=+16.17\;(C=2.3,\;{\mathrm{CHCl}}_{3})\;$$

Calculated (*m*/*z*): 474.2547 for C_26_H_38_O_4_N_2_S. Found: 474.2550.

**Figure 3:**
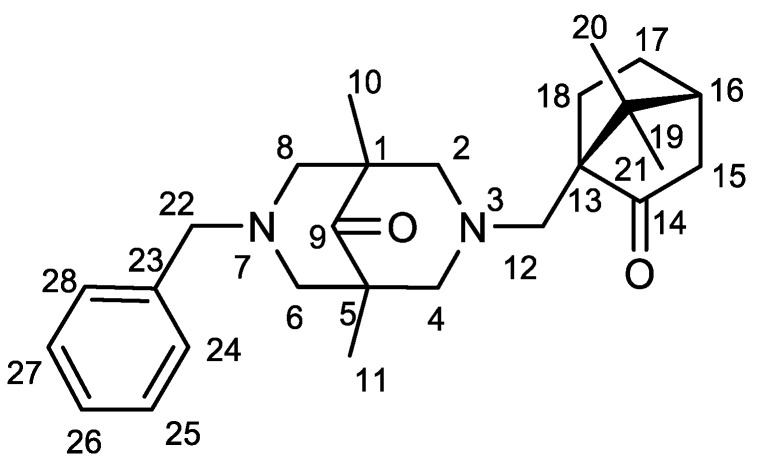
The structure of compound **37** with atom numbering scheme.

3-Benzyl-7-(((1*R*,4*R*)-7,7-dimethyl-2-oxobicyclo[2.2.1]heptan-1-yl)methyl)-1,5-dimethyl-3,7-diazabicyclo[3.3.1]nonan-9-one (**37**, Figure 3).

Bispidinone **24**, (100 mg (0.38 mmol)), 107 mg (0.38 mmol) of iodine derivative **31**, 160 mg (1.14 mmol) of anhydrous K_2_CO_3_ and 6 mL of CH_3_CN were added to the vial for the microwave reactor. The mixture was stirred until the organic substrates dissolved and put in the MW-reactor (temperature 75 °C, time 10 h). After completion of the reaction, the precipitate was separated, washed with EtOAc (2 × 10 mL). The organic phases were combined and evaporated. The resulting mixture of the desired product and starting compounds was purified by column chromatography on silica gel (2.5 g silica gel, eluent n-C_6_H_14_/EtOAc with the addition of 1 mL Et_3_N per 50 mL eluent). The desired product was isolated in an amount of 53 mg (34% yield).

^1^H NMR (600 MHz, CDCl_3_, δ, ppm, *J*, Hz; for the numbering of the atoms, see Figure 3): 0.76 (s, 3H, CH_3_) and 0.94–0.97 (3s, 9H) (C^10^H_3_, C^11^H_3_, C^20^H_3_, C^21^H_3_), 1.31–1.40 (m, 1H, C^13^H), 1.54–1.64 (m, 4H, C^19^H_3_, H-13), 1.75–1.83 (m, 2H, C^14^H, C^15^H), 2.21–2.29 (m, 1H, C^15^H’), 2.30–2.36 (m, 5H, skeleton C^2^H_2_, C^4^H_2_, C^6^H_2_, C^8^H_2_), 2.40 (d, 1H, *J* = 10.5, H-skeleton C^2^H_2_, C^4^H_2_, C^6^H_2_, C^8^H_2_), 2.97 (dd, 2H, *J* = 10.5, *J* = 3.8, C^12^H), 3.00 (d, 1H, *J* = 10.5, H-?) and 3.05 (d, 1H, *J* = 10.5) skeleton C^2^H_2_, C^4^H_2_, C^6^H_2_, C^8^H_2_),, 3.45–3.51 (2H, C^12^H12′), 5.21 (br.s, 1H, H-16), 7.20–7.25 (m, 1H, H-26), 7.26–7.34 (m, 4H, Ph).

^13^C NMR (151 MHz, CDCl_3_, δ, ppm): 46.41 and 46.52 (s, C1,C5), 56.14, 65.53, 65.61, 65.94 (t, C2, C4, C6, C8), 215.75 (s, C9), 20.06 (q, C10, C11), 61.07 (t, C22), 65.48 (t, C12), 27.3 (s, C13), 48.02 (d, C14), 35.47 (t, C15), 121.58 (d, C16), 148.49 (s, C17), 46.70 (s, C18), 12.51 (q, C19), 19.69 (q, C20, C21), 138.57 (s, C23), 126.93 (d, C26), 128.14 (d, C25, C27), 128.50 (d, C24, 28).

$${\left[\alpha \right]}_{D}^{24}\;=+25.0\;(C=0.66,\;{\mathrm{CHCl}}_{3})$$

Found [*M^+^*]: 408.2768. C_26_H_36_N_2_O_2_^+^. Calculated [*M^+^*]: 408.2771.

**Figure 4:**
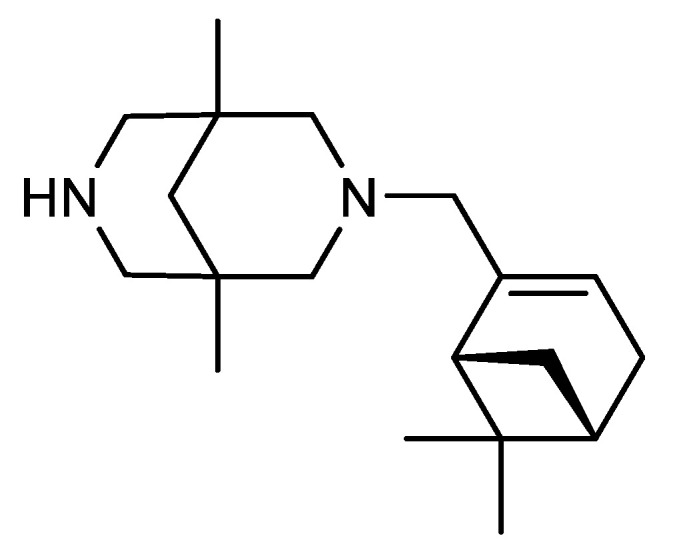
The structure of compound **38**.

3-(((1*R*,5*S*)-6,6-Dimethylbicyclo[3.1.1]hept-2-en-2-yl)methyl)-1,5-dimethyl-3,7-diazabicyclo[3.3.1]nonane (**38**, Figure 4).

214 mg (0.84 mmol) of amine **26**, 190 mg (0.88 mmol) of bromo-derivative **27**, 320 mg of K_2_CO_3_ and 5 mL of CH_3_CN were added to the vial for the microwave reactor. The mixture was stirred until the organic substrates dissolved and placed in the MW reactor (temperature 75 °C, time 2 h). After completion of the reaction, the potassium carbonate was filtered off, the reaction mixture was evaporated, and 320 mg of the mixture was obtained. According to gas chromatography–mass spectrometry (GC/MS), a mixture of mono- and di- alkylation products of the amine was formed, including a Boc-lacking product. Dissolution of the resulting mixture in EtOAc resulted in a white precipitate (53 mg). The residual mixture was separated on silica gel in a chloroform:ethanol system, but no other components of the mixture could be isolated individually.

^1^H NMR (400 MHz, CDCl_3_, δ, ppm, *J*, Hz): 0.78 (s, 3H, C^20^H_3_ or C^21^H_3_), 0.87 and 0.88 (s, 6H, C^10^H_3_, C^11^H_3_), 1.01 (d, 1H, *J* = 8.6, C^19^H^a^), 1.26 (s, 3H, C^20^H_3_ or C^21^H_3_), 1.30 (m, 1H, C^9^H), 1.46 (m, 1H, C^9^H’), 1.83 (dd, 1H, ^2^*J* = 11.6, *J* = 3.2, C^15^H), 1.96 (s, 1H, NH), 2.08 (m, 2H, C^16^H, C^18^H), 2.21–2.34 (dm, 3H, C^2^H, C^4^H, C^15^H’), 2.41 (m, 2H, C^2^H’, C^4^H’), 2.65 (dm, 1H, C^19^H), 2.84 (m, 2H, C^6^H, C^8^H), 2.93 (m, 2H, C^6^H’, C^8^H’), 3.10 (dm, 1H, C^12^H), 3.22 (dm, 1H, C^12^H), 5.31–5.35 (m, 1H, C^14^H).

^13^C NMR (100 MHz, CDCl_3_, δ, ppm): 24.73 (s, C1, C5), 55.31 (t, C6, C8), 64.80 (t, C2, C4), 44.25 (t, C9), 20.46 (q, C10, C11), 63.10 (t, C12), 136.98 (s, C13), 123.66 (d, C14), 30.47 (t, C15), 40.28 (d, C16), 37.28 (s, C17′), 44.21 (d, C18), 31.50 (t, C19), 26.01 (q, C20), 21.18 (q, C21).

$${\left[\alpha \right]}_{D}^{25}=+0.33\;(C=0.6,\;{\mathrm{CHCl}}_{3})\;$$

Found [*M^+^*]: 288.2564. C_19_H_32_N_2_^+^. Calculated [*M^+^*]: 288.2560.

**Figure 5:**
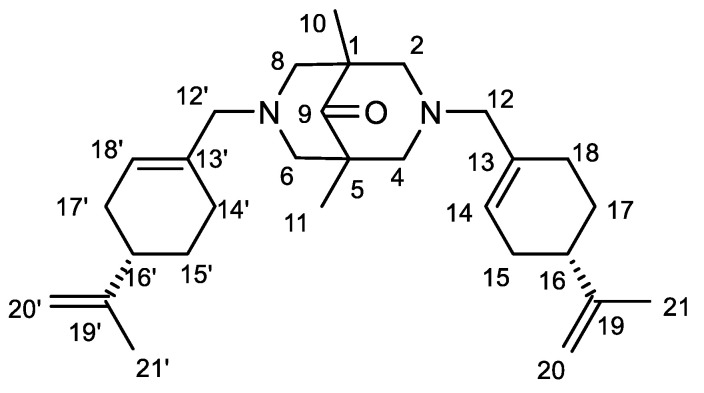
The structure of compound **42** with atom numbering scheme.

1,5-Dimethyl-3,7-bis(((*S*)-4-(prop-1-en-2-yl)cyclohex-1-enyl)methyl)-3,7-diazabicyclo[3.3.1]nonan-9-one (**42**, Figure 5).

A mixture of 476 mg (1.97 mmol) of bispidinonehydrochloride **16**, 2.5 eq of bromide **28** and 6 eq of K_2_CO_3_ in 4 mL acetonitrile was heated in a microwave reactor to 75 °C and allowed to stand for 90 min. After cooling of the reaction mixture, the precipitate was separated, washed with ethyl acetate. The organic phases were combined, the solvent was evaporated. The product yield after chromatography was 610 mg (71%).

^1^H NMR (600 MHz, CDCl_3_, δ, ppm, *J*, Hz; for the numbering of the atoms, see Figure 5): 0.96 s (6H, C10H3, C11H3); 1.39–1.47 m (2H, C^17^H^a^, C^17^′H^a−^); 1.72 br.s (6H, C^21^H_3_, C^21^′H_3_); 1.77–1.83 m (2H, C^17^H^e^, C^17′^H^e^); 1.88–2.03 m (4H, H-15a, H-15′a, H-18e, H-18′e); 2.07–2.16 m (6H, H-15e, H-15′e, H-16, H-16′, H-18a, H-18′a); 2.74 d (2H, ^2^*J* = 12.5, H’-12, H’-12′); 2.79 d (2H, ^2^*J* = 12.5, H’-12, H’-12′); 2.23 d (4H, ^2^*J* = 11.0); 2.86 dd (2H, ^2^*J* = 11.0, ^2^*J* = 1.2), 2.89 dd (2H, ^2^*J* = 11.0, ^2^*J* = 1.2)—4N-CH_2_; 4.67–4.69 m (2H, H’-20, H’-20′); 5.53–5.56 m (2H, H-14, H-14′).

^13^C NMR (151 MHz, CDCl_3_, δ, ppm): 46.38 s (C1, C5); 65.43 t, 65.50 t (C2, C4, C6, C8); 215.87 s (C9), 20.23 q (C10, C11); 63.74 t (C12, C12′); 135.10 s (C13, C13′); 123.86 d (C14, C14′); 30.51 t (C15, C15′); 41.14 d (C16, C16′); 27.58 t (C17, C17′); 27.46 d (C18, C18′); 149.76 t (C19, C19′); 108.44 q (C20, C20′); 20.71 q (C21, C21′).

$${\left[\alpha \right]}_{D}^{25.6}=-37.594\;(C=0.532,\;\mathrm{MeOH}).$$

Found [*M^+^*]: 436.6844. C_20_H_39_N_3_O^+^. Calculated [*M^+^*]: 436.6840.

## Figures and Tables

**Table 1:** The effect of the solvent on the yield of **15** in the presence of bispidinone **42** at −6 °C for 4 h.

Solvent	Yield, %	Conversion, %
Hexane	20	56
Diethyl ether	0	0
Dichloromethane	43	84
Acetonitrile	63	>98

**Table 2:** The effect of the reaction temperature (4 h) in MeCN on the conversion of the starting chalcone **3** in the presence of bispidinone **42**.

Temperature, °C	Conversion %
−20	77
−6	>98
4	100
22	100

**Table 3:** Adjustment of the amount of ligand to be used, using bispidinone **42** as an example (−6 °C, 4 h, MeCN).

Amount of the Diamine 42, mol.%	Ratio of Compounds 15 and 44 (15/44), GC/MS Data
8%	1
16%	1
24%	2.7

**Table 4:** Content of compounds **3**, **15** and **44** in reaction mixtures after 15, 30 and 60 min from the start of the reaction.

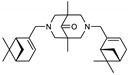	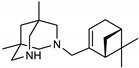
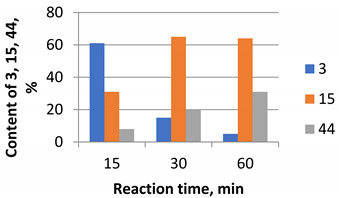	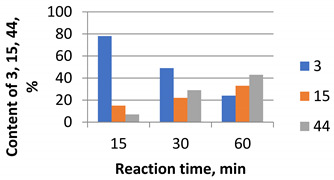
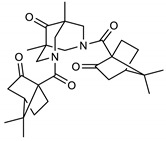 **35**	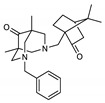 **37**
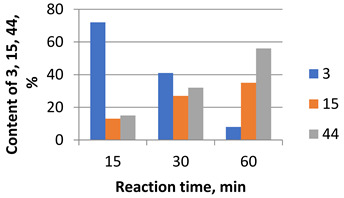	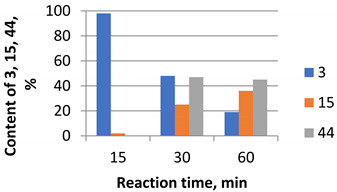
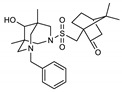 **36**	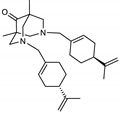 **42**
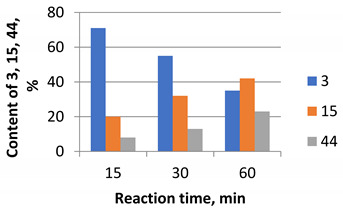	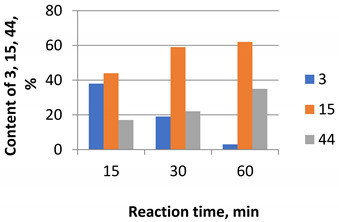

**Table 5:** Content of compounds **45**, **46** and **47** in reaction mixtures after 15, 30 and 60 min from the start of the reaction.

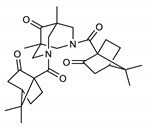 **35**	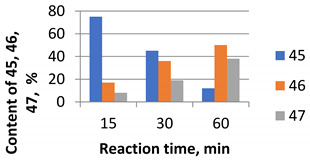
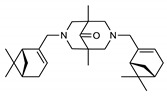 **43**	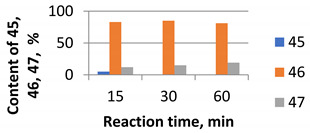
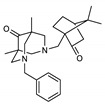 **37**	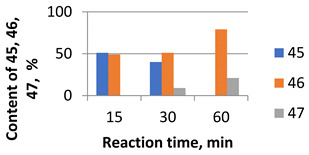

## Data Availability

Not applicable.

## Supplementary Materials

The following are available online. Figures S1–S8—NMR spectra of compounds; Figure S9—chromatogram of the reaction mixture.

## Sample Availability

Not available.
