# Supplementary material for: Novel Bispidine-Monoterpene Conjugates—Synthesis and Application as Ligands for the Catalytic Ethylation of Chalcones
Source: Molecules. 2021 Dec 13;26(24):7539. doi: 10.3390/molecules26247539 (PMC8703799; doi:10.3390/molecules26247539)

Novel bispidine-monoterpene conjugates - synthesis and application as ligands for the catalytic ethylation of chalcones

E.V. Suslov[1], K.Yu. Ponomarev[1], O.S. Patrusheva[1], S.O. Kuranov[1], A.A. Okhina[1,2], A.D. Rogachev[1], A.A. Munkuev[1], R.V. Ottenbacher[3], A.I. Dalinger[4], M.A. Kalinin[4], S.Z. Vatsadze[4*], K.P. Volcho[1*], N.F. Salakhutdinov[1],

[1]N.N. Vorozhtsov Novosibirsk Institute of Organic chemistry SB RAS, Lavrent'ev av., 9, 630090, Russian Federation, Novosibirsk

[2]Novosibirsk State University, Pirogov str., 2, 630090, Russian Federation, Novosibirsk

[3]Boreskov Institute of Catalysis SB RAS, Lavrent'ev av., 5, 630090, Russian Federation, Novosibirsk

[4]Chemistry Department, Lomonosov Moscow State University, Leninskie Gory, MSU, 1-3, 119991,Russian Federation, Moscow

Supplementary materials

Figure S1. ^{1}H NMR spectrum of compound **35**

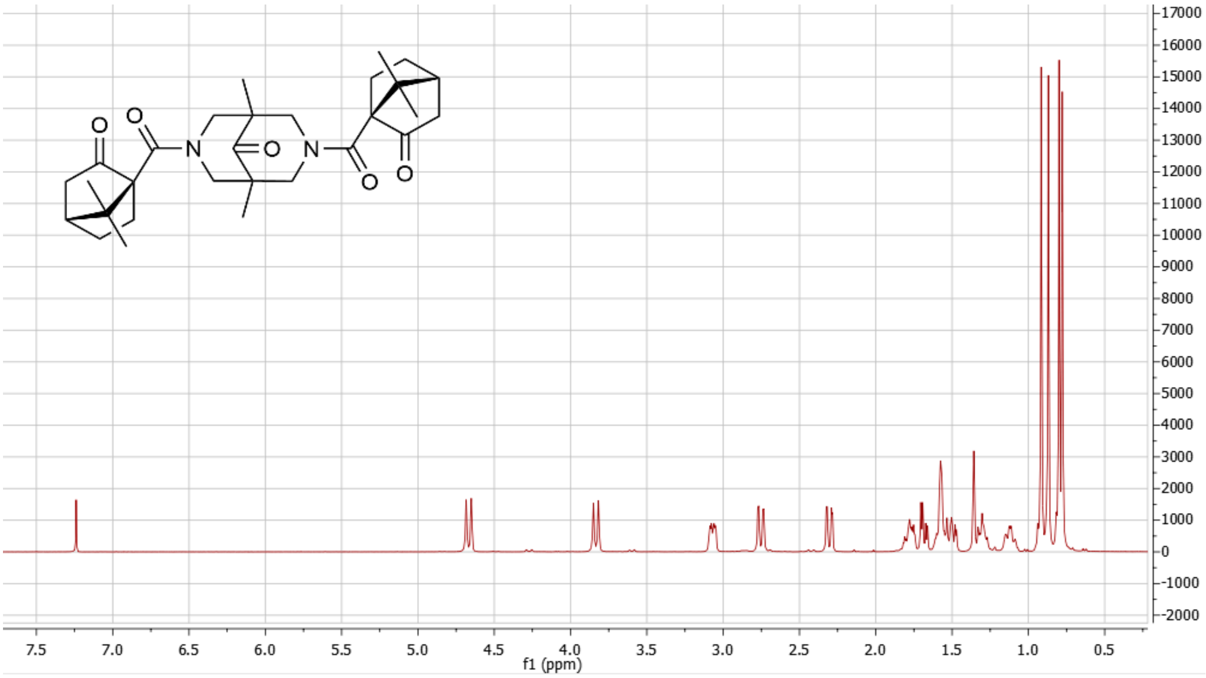

Figure S2. ^{13}C NMR spectrum of compound **35**

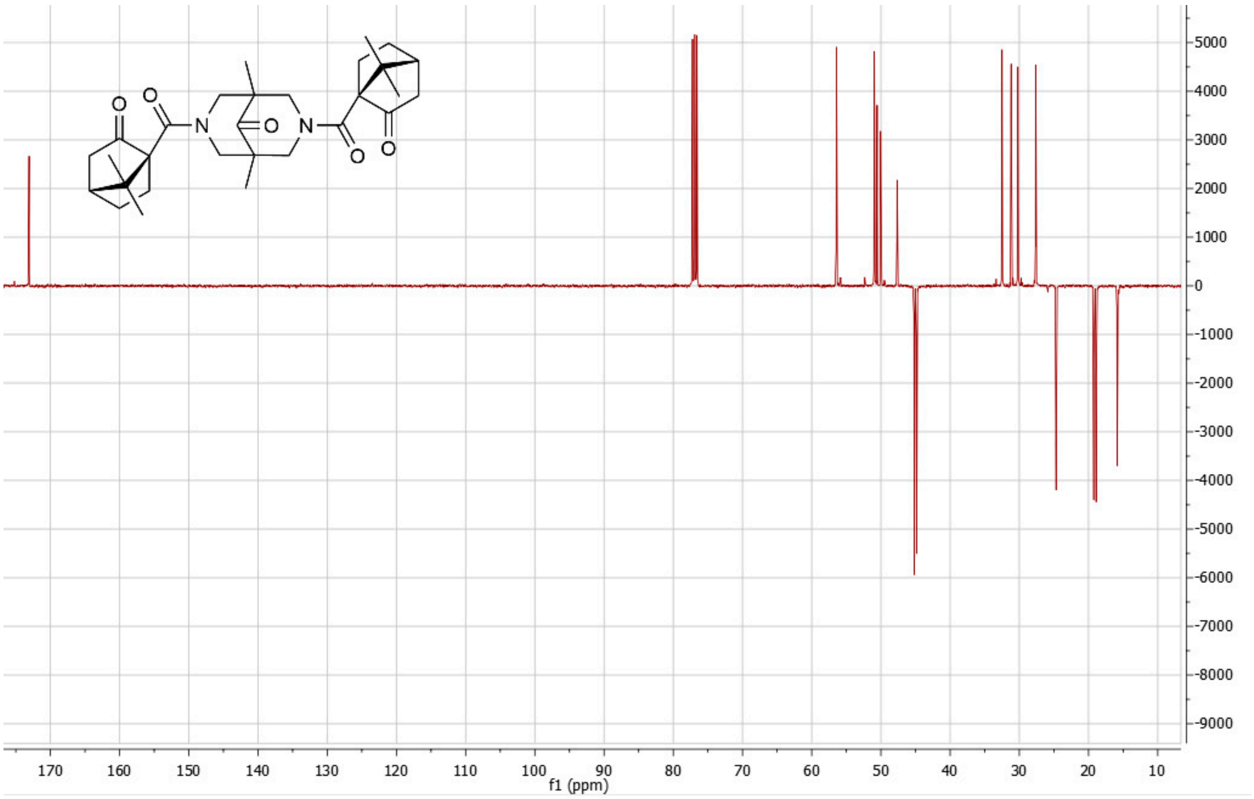

Figure S3. ^{1}H NMR spectrum of compound **36**

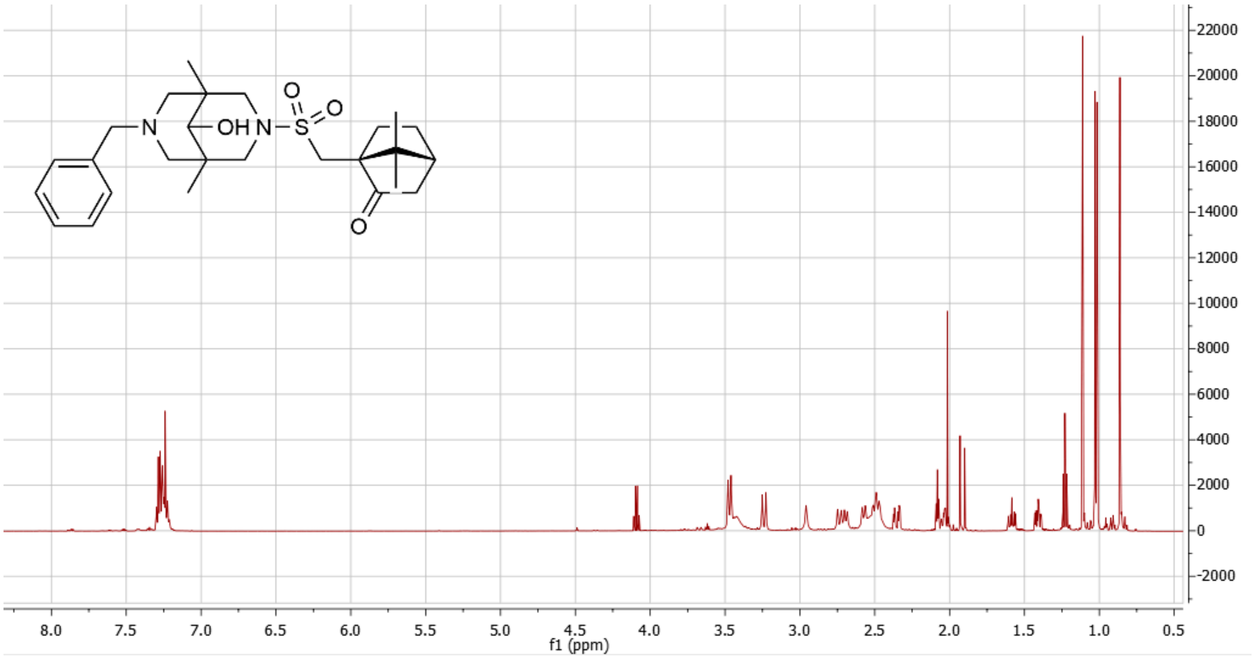

Figure S4. ^{13}C NMR spectrum of compound **36**

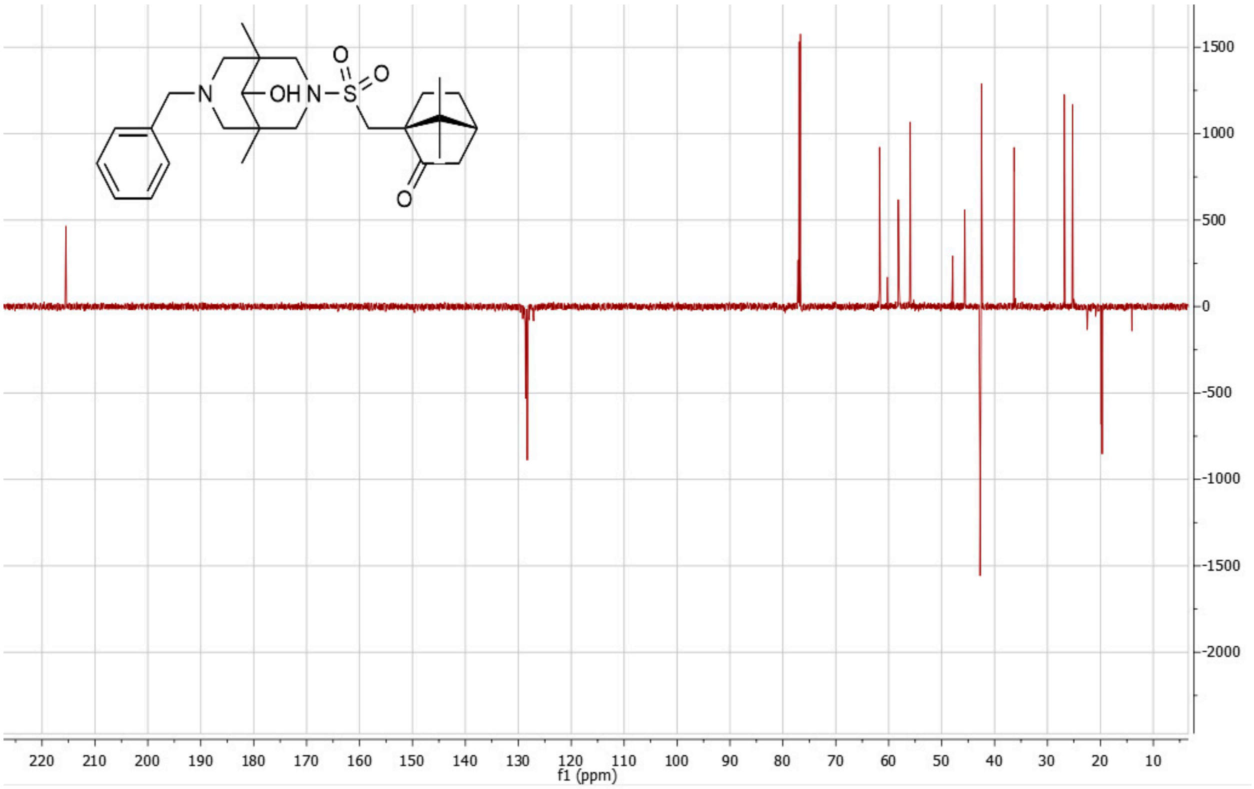

Figure S5. ^{1}H NMR spectrum of compound **37**

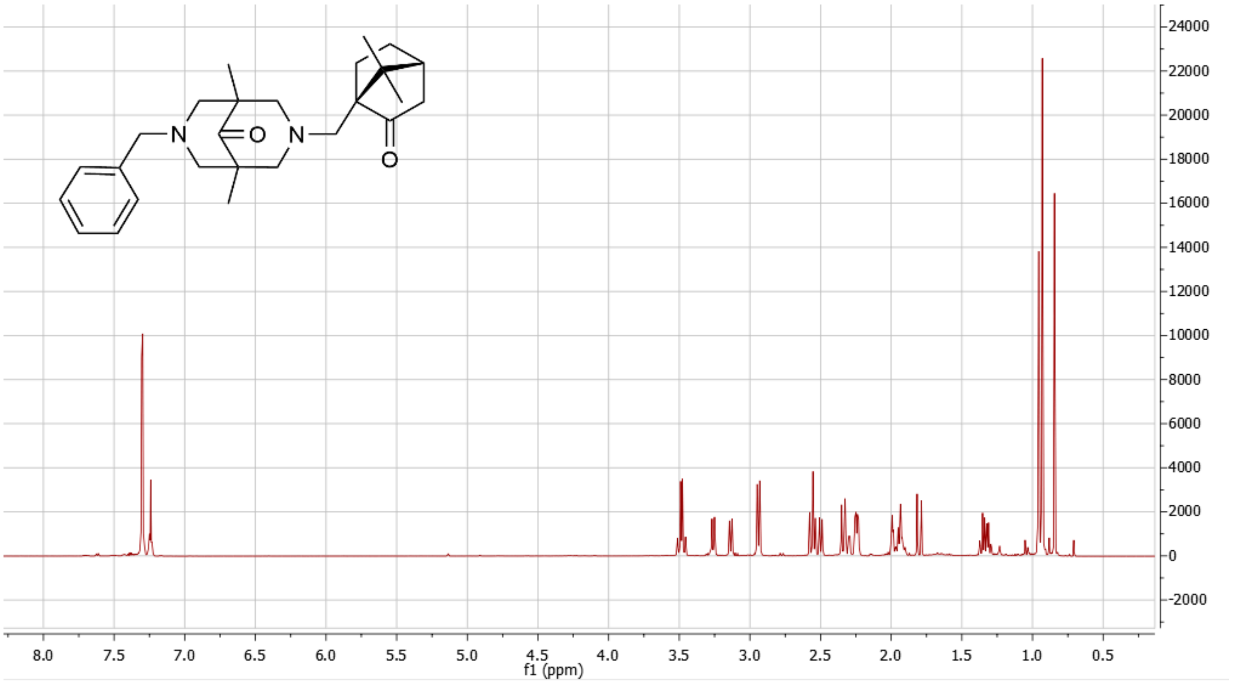

Figure S6. ^{13}C NMR spectrum of compound **37**

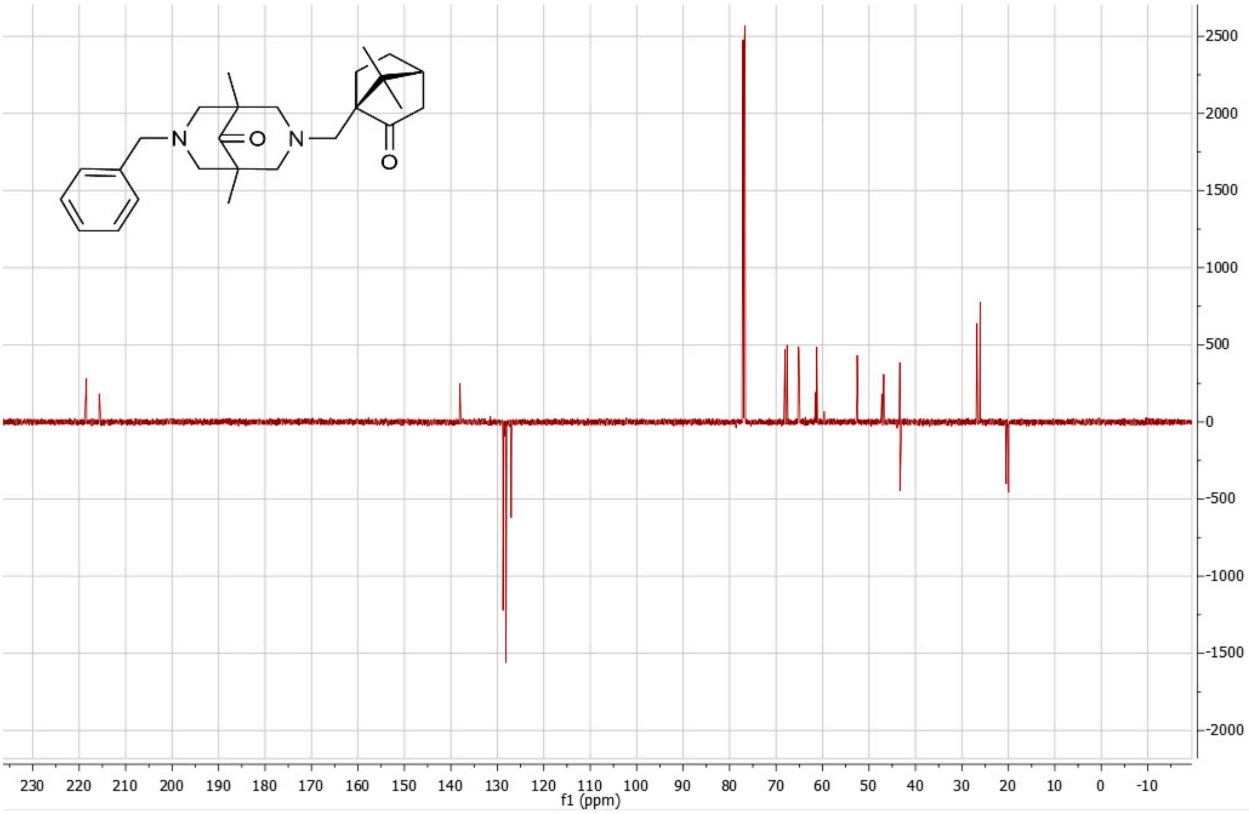

Figure S7. ^{1}H NMR spectrum of compound **42**

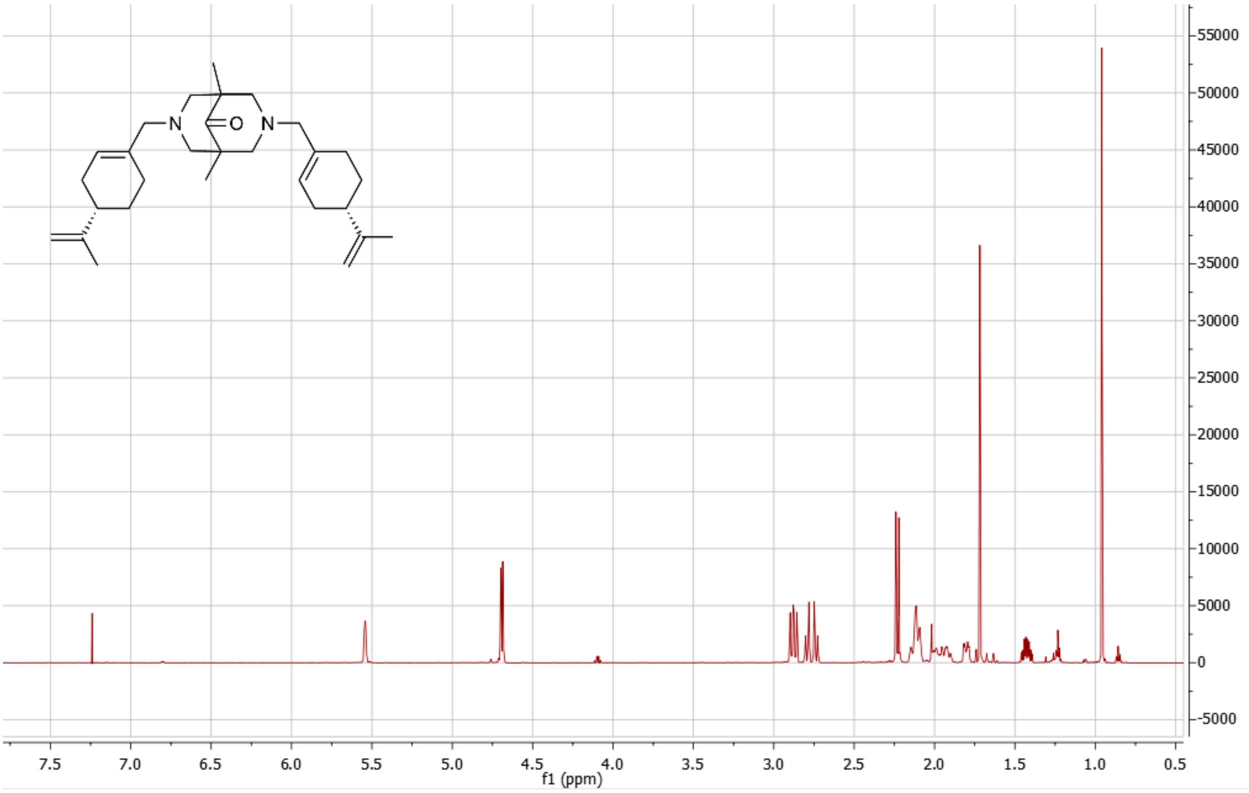

Figure S8. ^{13}C NMR spectrum of compound **42**

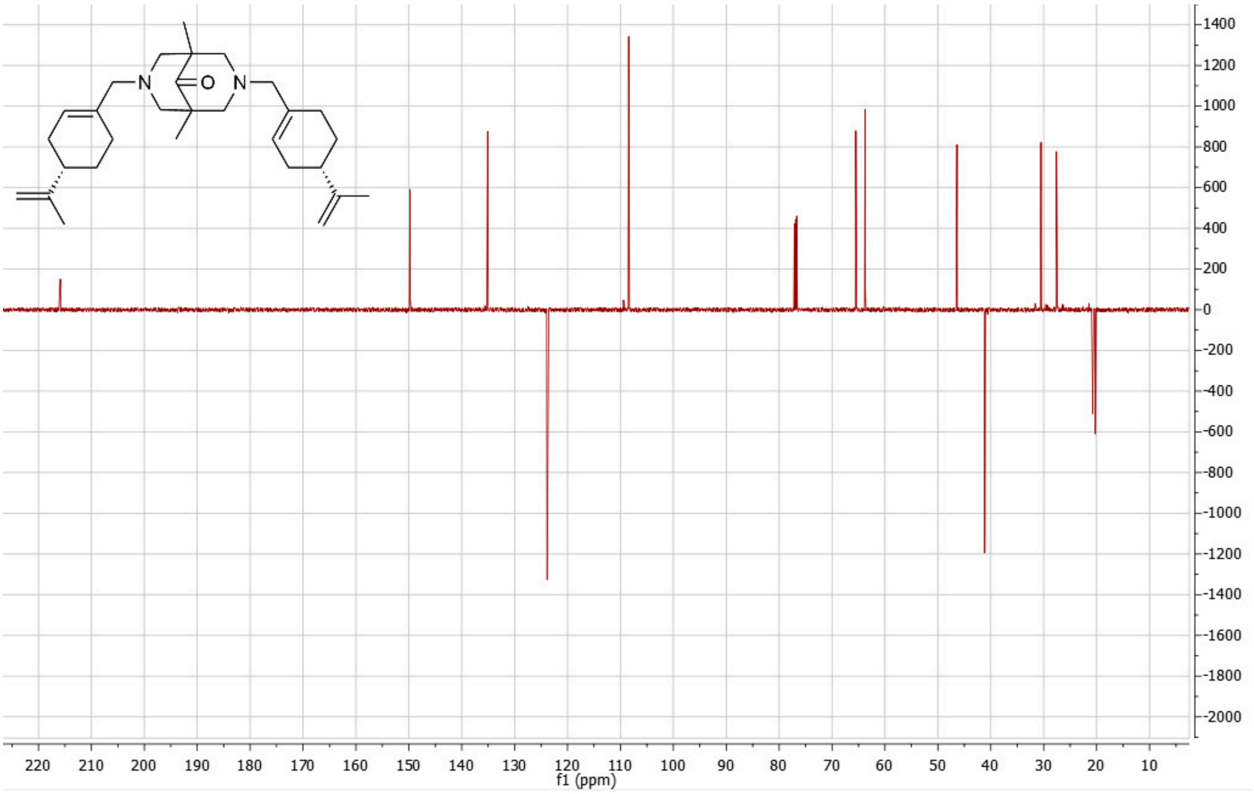

Figure S9. Chromatogram of the reaction mixture containing 1,3-diphenylpentane-1-one **15** (peaks at 7.2 and 7.9 min), 1,3-diphenylpropane-1-one **44** (peak at 10.7 min) and the starting chalcone **3** (peak at 11.5 min).

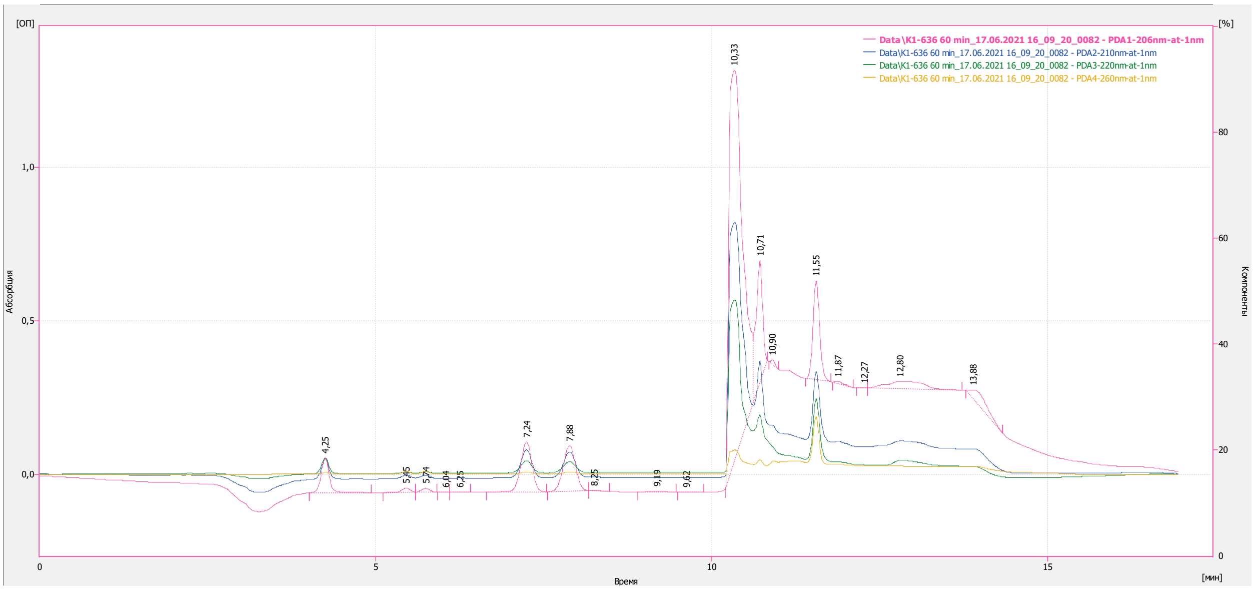

Supplement: Supplementary file 1 [file molecules-26-07539-s001.zip › molecules-1506151-supplementary.pdf]
